# Pharmacokinetic Characterization and External Evaluation of a Quantitative Framework of Sublingual Buprenorphine in Patients with an Opioid Disorder in Puerto Rico

**DOI:** 10.3390/pharmaceutics12121226

**Published:** 2020-12-18

**Authors:** Darlene Santiago, Victor Mangas-Sanjuan, Kyle Melin, Jorge Duconge, Wenchen Zhao, Raman Venkataramanan

**Affiliations:** 1 Pharmaceutical Sciences Department, School of Pharmacy, University of Puerto Rico, 00936-5067 San Juan, Puerto Rico; jorge.duconge@upr.edu; 2 Department of Pharmacy and Pharmaceutical Technology and Parasitology, University of Valencia, 46100 Valencia, Spain; victor.mangas@uv.es; 3 Interuniversity Research Institute for Molecular Recognition and Technological Development, Polytechnic University of Valencia, University of Valencia, 46100 Valencia, Spain; 4 Pharmacy Practice Department, School of Pharmacy, University of Puerto Rico, 00936-5067 San Juan, Puerto Rico; kyle.melin@upr.edu; 5 Department of Pharmaceutical Sciences, School of Pharmacy, University of Pittsburgh, Pittsburgh, PA 15261, USA; wez31@pitt.edu (W.Z.); rv@pitt.edu (R.V.); 6 Department of Pathology, MWRI, UPCI, MIRM, Thomas Starzl Transplantation Institute, School of Medicine, University of Pittsburgh, Pittsburgh, PA 15261, USA

**Keywords:** opioid use disorder (OUD), Suboxone, buprenorphine/naloxone sublingual film, Puerto Ricans, pharmacokinetics, popPK, population pharmacokinetics, compartmental modeling

## Abstract

Background: The aim of this analysis was to characterize the pharmacokinetics (PK) of sublingual buprenorphine (BUP) and its metabolites (buprenorphine glucuronide; BUP-g, norbuprenorphine; Nor-BUP, and norbuprenorphine glucuronide; Nor-BUP-g) in opioid use disorder (OUD) patients in Puerto Rico (PR) as a first step of evidence-based BUP dosing strategies in this population. Methods: BUP and metabolites concentrations were measured from 0 to 8 h after the administration of sublingual buprenorphine/naloxone films in 12 stable OUD subjects. Results: PK non-compartmental characteristics showed considerable variability in parameters between the subjects over the 8-h sampling time (*t_max_* = 1.5 ± 0.7 h, *C_o_* = 1.6 ± 1.4 ng/mL, *C_max_*= 7.1 ± 6 ng/mL, and *AUC*_0–8h_ = 26.8 ± 17.8 h·ng/mL). Subjects had a significantly higher tendency towards CYP-mediated *N*-demethylation, with the *AUC*_0–8h_ ratios of the molar concentrations of [Nor-BUP + Nor-BUP-g] to BUP being (3.4 ± 1.9) significantly higher compared with BUP-g to BUP (0.19 ± 0.2). A two-compartment population-PK model with linear absorption (*k*_a_ = 2.54 h^−1^), distribution (*k*_12_= 2.34 h^−1^, *k*_14_ = 1.29 h^−1^), metabolism (*k*_24_ = 1.28 × 10^−1^ h^−1^, *k*_23_ = 6.43 × 10^−2^ h^−1^, *k*_35_ = 1.23 × 10^−1^ h^−1^, *k*_45_ = 8.73 × 10^−1^ h^−1^), and elimination (*k*_30_ = 3.81 × 10^−3^ h^−1^, *k*_50_ = 1.27 × 10^−1^ h^−1^) adequately described the time-course of BUP and its metabolites, which has been externally validated using published data. Conclusions: Although limited in sampling time and number of recruited subjects, this study presents specific BUP PK characteristics that evidenced the need for additional PK studies and subsequent modeling of the data for the development of evidence-based dosing approaches in Puerto Rico.

## 1. Introduction

Opioid Use Disorder (OUD) is an unprecedented disease both in Puerto Rico (PR) and the greater United States (US), with an increase in the number of opioid users every year [1]. In 2017, 600 opioid overdoses were reported in PR, which was 400 more than the previous year [2]. Buprenorphine (BUP) is considered a safe pharmacotherapy for OUD in PR and the US due to its partial agonistic characteristics, high μ-opioid receptor (μOR) affinity, longer half-life (when administered sublingually) and its ceiling effect that results in a plateau of opioid agonistic effects [3]. Sublingual administration of BUP for OUD has demonstrated higher bioavailability compared to oral administration, with a peak serum concentration reached 1.5–1.7 h after single film administration (*t_max_*), and a half-life (*t*_1/2_) of 33.44–32.82 h [4]. Current guidelines in the US for OUD recommend a combination of pharmacologic maintenance treatment (usually with BUP or methadone) and psychosocial treatment [5,6]. In PR, the dosing approach for OUD pharmacotherapy using BUP or methadone is an extrapolation of the treatment protocols developed in the US. The use of methadone is often limited to specific federal and state-licensed facilities, whereas BUP is more accessible through regular outpatient prescriptions filled in a community pharmacy [5,6,7]. This increased accessibility of BUP coupled with the growing number of patients seeking treatment for OUD in PR has led to a significant increase in the number of BUP prescriptions, even as methadone use has decreased [8].

Clinical efficacy of BUP for OUD is based on its ability to occupy the *μ*OR in the brain and to suppress the withdrawal symptoms, i.e., agonistic substitution. Increasing the dose of BUP, and hence, plasma levels, results in higher agonistic substitution of the *μ*OR and higher clinical efficacy. Clinical studies have elucidated *μ*OR saturation to be associated with BUP plasma levels and dosing required for suppression of withdrawal symptoms and blockage of opioid reinforcement [9,10]. While suppression of withdrawal symptoms requires blockage of >50% *μ*OR (<50% *μ*OR availability) associated with plasma BUP concentrations of ≥1 ng/mL and daily dose of 4 mg, higher blockade (>80% *μ*OR) is required for suppression of opioid reinforcement effects of typical doses of most illicit drugs, associated with BUP plasma levels of ≥3 ng/mL, and daily BUP dose of 16 mg [11]. Population pharmacokinetic (popPK) BUP models have reported dose adjustments in newborns with neonatal abstinence syndrome (NAS) [12]. In addition, a popPK modeling strategy has also been reported with predominantly Whites subjects to predict μOR occupancy given a determined BUP dose from a novel sustained release BUP formulation [13]. To our knowledge, this last model was the first to scientifically determine a BUP dose range that would sustain specific μOR occupancy for the suppression of withdrawal symptoms.

Although similar BUP PK profiles have been reported between different population groups [14,15,16], variability in the pharmacokinetics of its metabolites and its corresponding PK profiles reflects differences in BUP metabolic pathways between groups (activity and expression of UGT and CYP), which may put some population groups at higher risk for treatment failure. These differences emphasize even more the need for personalized dosing for BUP in different populations to optimize OUD therapy. The present analysis was conducted to characterize the pharmacokinetics of sublingual BUP (prescribed as buprenorphine/naloxone films; Suboxone^®^) in stable maintenance phase OUD patients in PR, and to develop a preliminary population PK model as a first step of evidence-based BUP dosing strategies in this population.

## 2. Materials and Methods

### 2.1. Participants and Procedures

Institutional review board approval for this study was obtained in October 2017 (IRB#B1080117) by the campus board, and the study started recruitment in November 2017. Written informed consent was obtained from all participants prior to the study.

Twelve OUD subjects were recruited for this study: six males and six females. Patients in their maintenance phase and prescribed sublingual formulation Suboxone^®^ (buprenorphine/naloxone) for a minimum of three months were eligible to participate in the study. Although patients were prescribed once a day dosing, most of the patients were using twice daily dosing by dividing their daily dose of their own volition. However, on the day of the study, patients were required to take their entire prescribed daily dose in front of the researchers. Other inclusion criteria included 21 years of age or older (the age of majority in PR), with their parents and grandparents being born and raised and having lived in Puerto Rico. Additionally, the participants must have lived in Puerto Rico for at least half of their lifetime. Participants were excluded if they were unable to consent, and if on the day of the study they tested positive for illicit opioid use. Upon signing the consent forms, subjects were scheduled to come to the clinic on a separate day for the pharmacokinetic sampling. On the day of the pharmacokinetic sampling, participants were asked to provide a urine sample to test illicit opioid use. If negative, the participants were asked to provide immediately one blood sample prior to taking their daily dose of Suboxone^®^ sublingual. Additional blood samples were then drawn at 0.5, 1, 2, 3, 4, 6 and 8 h after the dose and collected using lavender EDTA tubes. Whole blood samples were centrifuged at 3000 rpms for 10 min to obtain plasma. The plasma was stored in cryo-freezing vials at −80 °C for further analysis.

### 2.2. Assay Methodology

Plasma concentrations of BUP, Nor-buprenorphine (Nor-BUP), Buprenorphine-3-glucuronide (BUP-g) and Norbuprenorphine-3-glucuronide (Nor-BUP-g) were determined by Ultra Performance Liquid Chromatography—Mass Spectrometry (UPLC-MS/MS), which was developed and validated in our laboratory following FDA guidance for analytical procedures and methods validation for drugs and biologics [17]. Buprenorphine-D_4,_ norbuprenorphine-D_3_, buprenorphine-D_4_-3-β-D-glucuronide, and norbuprenorphine glucuronide-D_3_ were used as internal standards. The UPLC system consisted of Waters Acquity H class and the mass spectrometric analysis was carried out using a XEVO TQS triple quadrupole mass spectrometer (Waters, Milford, MA, USA) with positive electric spray ionization mode using multiple reaction monitoring (MRM) mode. The QC plasma samples at four different concentrations were processed to examine the accuracy and precision of the assay. The intra-day and inter-day accuracies expressed as percentage of the nominal concentrations were within 98.2–108.0%. The intra-day and inter-day precision determined by the coefficient of variations were within 9%. The standard curve was linear from 0.05 ng/mL to 100 ng/mL for all the analytes. The intraday and interday coefficient of variation was less than 10% for all the analytes.

### 2.3. Non-Compartmental Pharmacokinetic Analysis

Pharmacokinetic parameters of all subjects were estimated from individual concentration-time data by non-compartmental analysis using Phoenix 32^®^ WinNonlin^®^ software (version 8.1.0, 2018; Certara Inc., Princeton, NJ, USA). Estimated parameters included the maximum concentration (*C_max_*), time for maximum concentration (*t_max_*) and area under the curve (*AUC*_0–8h_). The first blood sample before dosing was taken as the trough of the plasma concentration (*C*_0_).

Reported observed *AUC*_0–8h_ values correspond to the 8 h sampling time. Given the long half-life of BUP (approximately 37 h), and the 8 h sampling interval of the study, it was not possible to estimate the half-life and the *AUC* from time zero to infinity (*AUC*_0–∞_).

The trapezoidal rule was used to obtain *AUC* ratios to estimate the BUP CYP- and UGT-mediated metabolic pathways; these were then converted to molar *AUCs* (h mol/mL) [18]. The molar *AUC* ratios of Nor-BUP and Nor-BUP-g to BUP were used as a measure of CYP3A4 mediated *N*-demethylation pathway. The molar AUC ratio of BUP-g to BUP was used as an indicator of the UGT pathway.

### 2.4. Population Pharmacokinetic Analysis

A pilot pharmacokinetic analysis was preliminary performed to describe the estimating capabilities of a model developed with 12 subjects. A quantitative mathematical framework was developed as a first step in characterizing the time-course of BUP, Nor-BUP, BUP-g and Nor-BUP-g in recruited Puerto Rican subjects with compartmental models parameterized in first order rate constants and apparent volumes of distribution. In order to avoid identifiability issues, the apparent volume of distribution of Nor-BUP was assumed to be equal to BUP (*V*_2_), and a different apparent volume of distribution (*V*_3_) for BUP-g and Nor-BUP-g was defined. The metabolism of BUP was described through sequential, simultaneous and liver-like compartment processes in order to simultaneously characterize the PK of all the analytes.

All data analyses were performed based on the population approach with the software NONMEM^®^ (v7.3, 2013, ICON plc Development Solutions, Hanover, MD, USA). Plasma concentrations were logarithmically transformed. The population PK parameters were estimated using the Stochastic Approximation of the Expectation Maximization and the Importance Sampling Estimation method. Inter-individual variability (IIV) associated to the PK model parameters was modeled exponentially, preventing negative values of the individual estimates, and residual unexplained variability (RUV) was described with an additive model on the logarithmic scale. The significance of the non-diagonal elements of the Ω variance-covariance matrix and subject specific RUV were also evaluated.

Model selection was established through the minimum value of the objective function value (OFV) provided by NONMEM^®^, which was approximately equal to −2 × log(likelihood) (−2LL), together with the visual inspection of the goodness of fit (GOF) plots. A decrease of 3.84 points of the −2LL value between two nested models differing in one parameter was considered significant at the 5% level. Evaluation of the selected models was performed through prediction-corrected visual predictive checks (pc-VPC) [19]. Briefly, one thousand simulated datasets were simulated, and the 2.5th, 50th, and 97.5th percentiles for every simulated study and sampling time period were calculated. Then, the 95% prediction intervals of the above-described percentiles were calculated and displayed graphically together with corresponding percentiles computed from raw data. Precision of the model parameter estimates, defined as the relative standard error (RSE), was calculated from the variance-covariance matrix (when possible) and from the analysis of one thousand simulated bootstrap datasets.

For graphical and statistical analysis, R software (http://cran.r-project.org, version 3.5.0, 2018) was used. Pc-VPC and bootstrap analysis were performed using PsN [20].

## 3. Results

The average age and weight of the recruited participants was 46.7 years and 151 lbs (68 kg), respectively. All 12 participants provided consent to participate in the study. The day of the study all participants tested negative for illicit opioid use, and all were maintained at steady BUP dose for at least three months prior to the study using Suboxone^®^. Nine participants adhered to the eight hours of pharmacokinetic sampling; among the participants, eight blood samples were drawn from seven participants, and seven samples were drawn from four participants. Plasma concentrations of one male participant had to be excluded from the analysis due to noticeable BUP plasma concentration fluctuations, which the investigators suspect was the result of additional self-administration of BUP by the participant during the study, unnoticed by the investigators. This resulted in a total of 84 plasma samples from 11 subjects for pharmacokinetic characterization and data analysis.

### 3.1. Non-Compartmental PK Analysis

**Figure 1:**
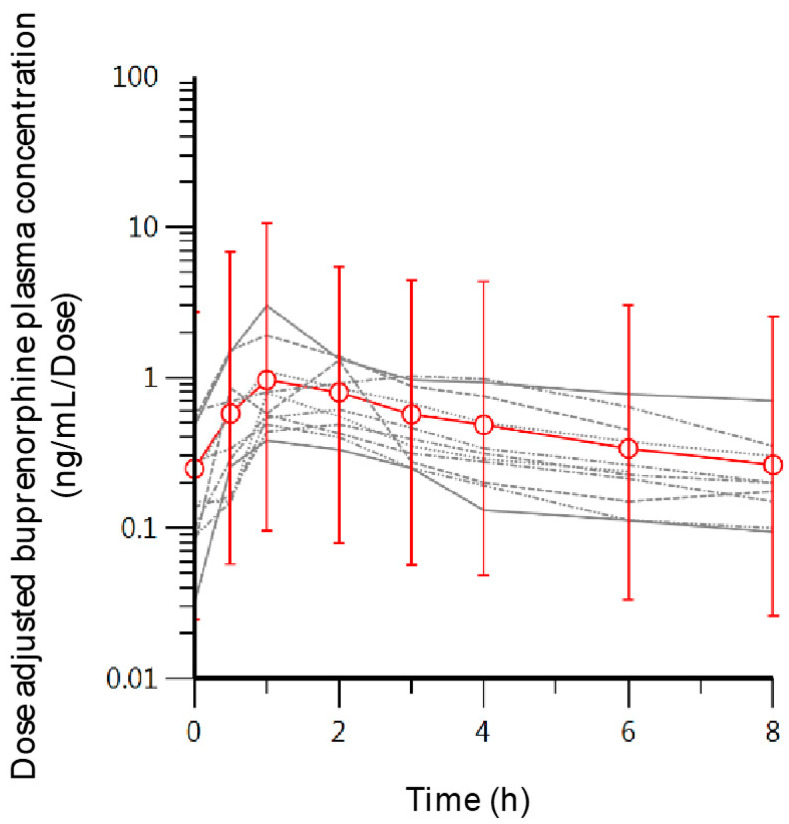
Individual concentration-time curves (grey lines) and the mean concentration at each sampling point (red open circles) with the standard deviation as error bars.

The average prescribed maintenance dose of the participants was 7.6 ± 3.3 mg of BUP (Table 1). Pharmacokinetic parameters observed estimates were: *t_max_* = 1.5 ± 0.7 h, *C_o_* = 1.6 + 1.4 ng/mL, *C_max_* = 7.2 ± 6 ng/mL, *AUC*_0–8h_ = 26.8 ± 17.8 ng·h/mL. Dose-adjusted plasma concentration of all participants increased rapidly upon dosing, up to an average dose-adjusted maximum concentration (*C_max_*) of 1.06 ± 0.79 ng/mL/mg (Table 1; Figure 1). There was considerable variability in the concentration versus time curves between subjects, as observed in the high relative standard deviation percentages of the estimated pharmacokinetic parameters, which ranged between 43% and 80% (Table 1). Dose-adjusted *C_max_* and *AUC*_0–8h_ were 1.06 ± 0.79 ng/mL/mg and 3.84 ± 2.3 ng·h/mL/mg, respectively (Table 1). Average maximum and minimum plasma levels of BUP and metabolites within the 8-h sampling time are shown in Table 2. The average maximum concentration of the metabolite Nor-BUP-g throughout the eight-hour sampling time was significantly higher compared to the plasma levels of BUP, Nor-BUP and BUP-g. This same behavior was observed with the minimum plasma concentration.

The recruited subjects had a significantly high tendency towards the CYP-mediated *N*-methylation pathway of BUP with *AUC*_0–8h_ ratios of Nor-BUP and Nor-BUP-g to BUP compared to *AUC*_0–8h_ ratios of BUP-g to BUP (Table 3).

**Figure 2:**
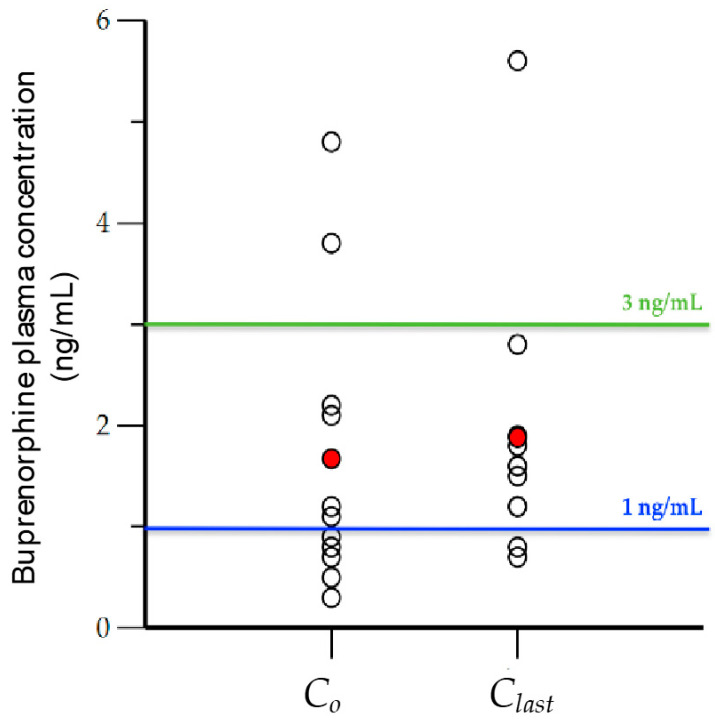
Initial (*C_o_*) and last (*C_last_*) observed buprenorphine concentration (ng/mL) drawn at times 0 and 8 h (open black circles), and the average value of each (red circles).

Figure 2 illustrates the BUP trough levels (*C_o_*) drawn just before dose administration and the last drawn blood levels (*C_last_*) of participants (open black circles), and their corresponding average values (red circles). The average values for *C_o_* and *C_last_*, although higher than 1 ng/mL (1.6 ng/mL and 1.8 ng/mL, respectively), were lower than 3 ng/mL. Five subjects, and possibly two more (total of seven) just before dosing had BUP blood levels (*C_o_*) below or near 1 ng/mL. This was also the case for two subjects, who had BUP blood levels <1 ng/mL 8 h after dosing (*C_last_*). A significant number of subjects had trough blood levels below 3 ng/mL throughout the sampling interval (8 h).

Although the BUP trough and 1 h blood levels had a weak correlation (*r* = 0.645 and 0.311) with BUP 8 h exposure (*AUC*_0–8h_), strong correlations existed between plasma levels measured 2 and 3 h after administration (*R*^2^ = 0.886 and *R*^2^ = 0.856, respectively) and exposure. This suggests drawing blood 2 or 3 h after administration may be an appropriate surrogate for *AUC*_0–8h_ when sampling is limited.

### 3.2. Population PK Analysis

**Figure 3:**
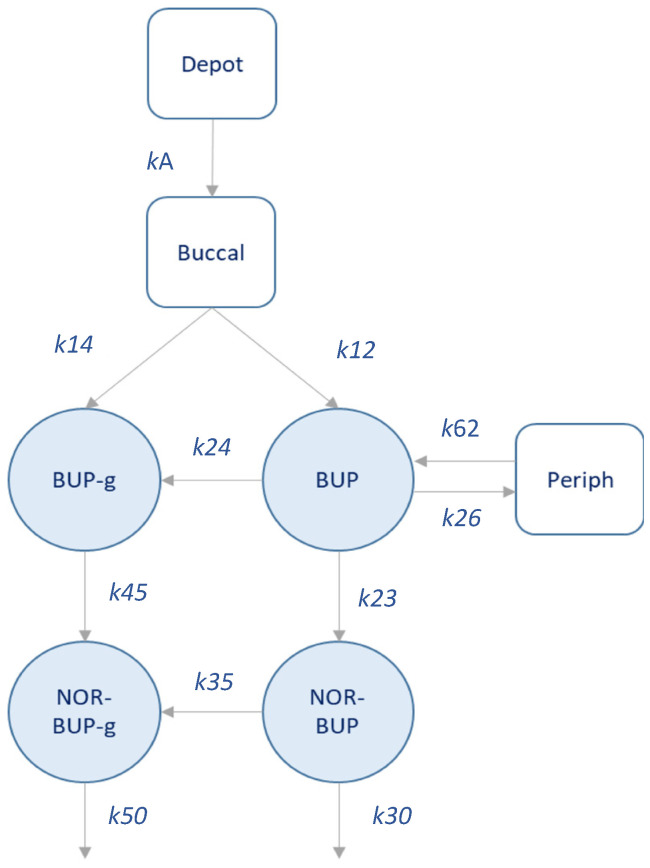
Schematic representation of the population PK model developed.

A total of 307 plasma levels of BUP, Nor-BUP, BUP-g and Nor-BUP-g from 10 patients were considered for the preliminary population PK analysis. Figure 3 depicts a schematic representation of the developed pilot BUP population model (popPK). The structural model assumes rapid absorption of BUP (*k*_a_ = 2.54 h^−1^) into a buccal compartment, from which a partial metabolism of BUP to BUP-g appear to occur (*k*_14_). The distribution of BUP into the central compartment occurs at a higher rate compared to BUP-g, since k_12_ is significantly higher than *k*_14_. Subsequently, BUP is distributed according to a two-compartment model. From the central compartment, BUP is metabolized to BUP-g (*k*_24_ = 1.28 × 10^−1^ h^−1^) and Nor-BUP (*k*_23_ = 6.43 × 10^−2^ h^−1^), in similar kinetic terms. Nor-BUP is mainly metabolized to Nor-BUP-g (*k*_35_ = 1.23 × 10^−1^ h^−1^) and, to a minor extent, is eliminated unchanged (*k*_30_ = 3.81 × 10^−3^ h^−1^). The transformation of BUP-g is carried out to Nor-BUP-g, which is eliminated from the body (*k*_50_ = 1.27 × 10^−1^ h^−1^).

**Figure 4:**
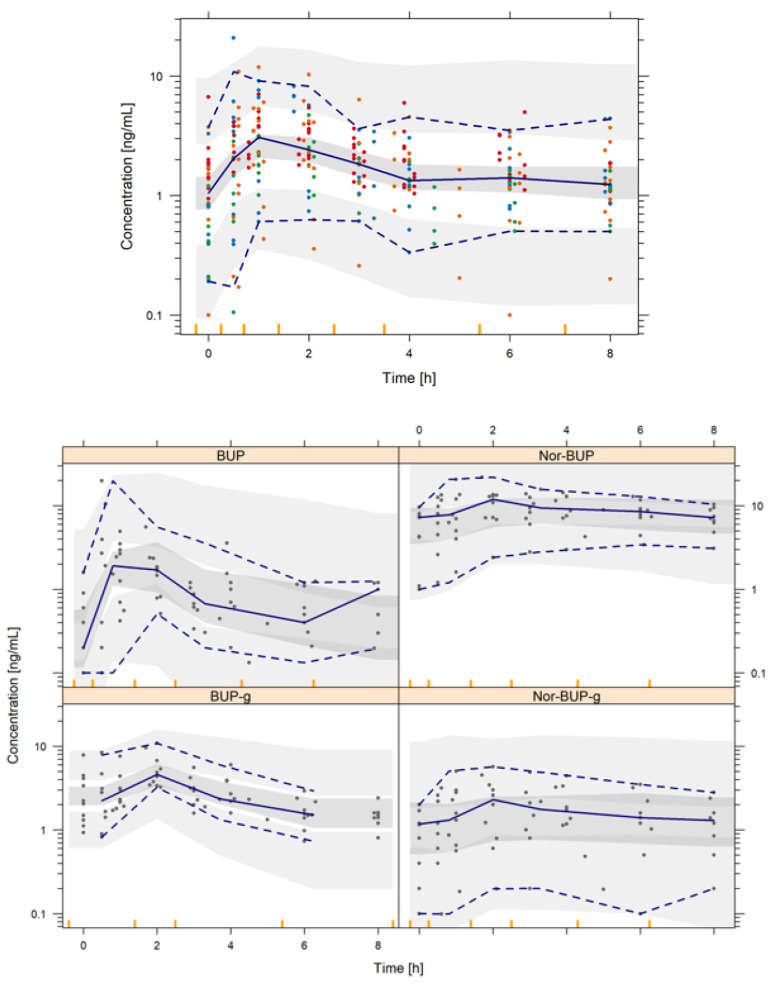
Prediction-corrected visual predictive check of the final population pharmacokinetic model. Shaded areas represent the 95% prediction intervals of the 2.5th, 50th and 97.5th percentiles of the simulated data. Points are the observed BUP (red), Nor-BUP (orange), BUP-g (green) and Nor-BUP-g (blue) concentrations. Lines represent the 2.5th, 50th and 97.5th percentiles of the raw data.

Table 4 summarizes the preliminary PK model parameter estimates, precision and bootstrap results. The adequacy of this initial model to capture the plasma levels of each analyte is represented in Figure 4. The analysis of the inter-individual variability has confirmed its significance in *k*_24_, *k*_23_, *k*_35_, *k*_45_ and *k*_50_ parameters, showing a moderate-to-high behavior. Non-diagonal elements were explored, but they were not statistically significant (*p* > 0.05). The developed preliminary popPK model predictions, represented as shaded areas (Table 4), demonstrate that although preliminary, this pilot model has the potential to describe the observed behavior of each analyte, both in its mean trend and in the observed variability. Standard GOF plots and individual model predictions are shown in the Supplementary Material Figures S1–S3. Table 5 summarizes the external validation of the popPK model developed through the comparison of model-predicted and observed *AUC* and *C_max_* values across several dose levels of single-dose regimen of BUP. The exposure values have been obtained by simulating a virtual population of 1000 patients, following the same study conditions. Subsequently, the *AUC* values were calculated using the trapezoidal rule and the *C_max_* value directly from the simulated values. Most of the ratios (88% for *AUC* and 97% for *C_max_*) for both exposure endpoints of BUP lay between 0.5–1.5, which indicates a good predictive capacity of the developed model.

## 4. Discussion

This study describes for the first time the pharmacokinetics of sublingual BUP (buprenorphine/naloxone Suboxone^®^ films) in a Puerto Rican population diagnosed with opioid use disorder (OUD), who are on a stable maintenance BUP dose. In addition, for the first time a preliminary BUP population PK (popPK) model is proposed for this specific population group. Our pharmacokinetic study describes considerable pharmacokinetic variability (also observed in other BUP PK studies) within recruited Puerto Rican OUD patients with dose-normalized values [16,26,28,29,30]. This was evident from the relative standard deviation of the estimated area under the curve (*AUC*_0–8h_) and maximum concentration (*C_max_*) values. Although the variability in this study is greater than desired, some population comparisons may be described. The time for maximum concentration (*t_max_*) of recruited Puerto Rican subjects was comparable to reported values from predominantly African American subjects maintained with an average dose of 16 mg [15]; however, they were higher than Chinese subjects, which ranged between 0.7–1 h maintained with a similar dose (8 mg) [14]. Absolute maximum plasma levels were slightly higher compared to these same groups; however, they were slightly lower compared to a study with predominantly White OUD subjects maintained at a higher dose (16 mg) of BUP [31]. Observed BUP eight-hour exposure (*AUC*_0–8h_) of recruited subjects was expected to be lower compared to the *AUC*_0–24h_ of these ethnic groups. The differences between these groups (African American, Chinese and White), however, suggests the need for additional pharmacokinetic evaluations of subjects in Puerto Rico with a larger sampling time to accurately assess exposure differences. Observed dose normalized *AUC*_0–8h_ was closer, however, to reported *AUC*_0–12h_ in predominantly White females (4.0 ± 2.5 ng·h/mL/dose) [16].

**Figure 5:**
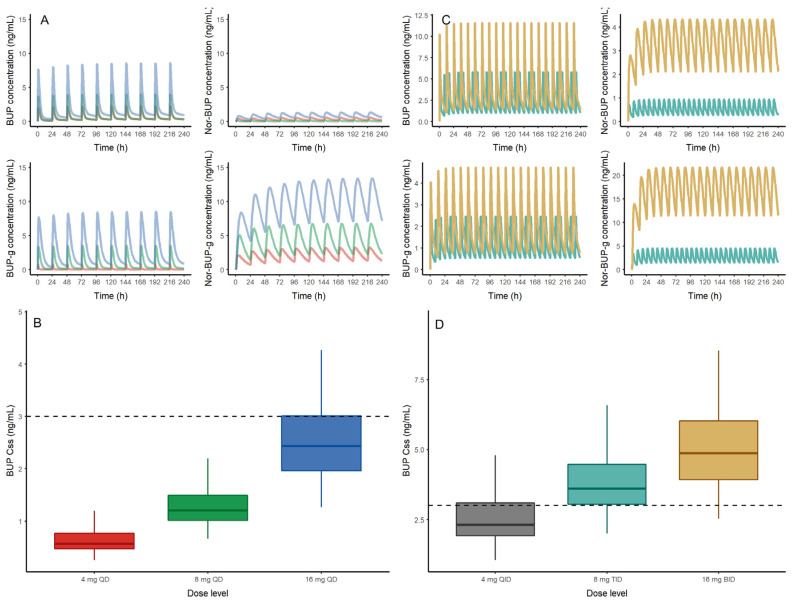
(**A**) (Top) Deterministic simulation of buprenorphine (BUP), nor-buprenorphine (Nor-BUP), buprenorphine-glucuronide (BUP-g) and nor-buprenorphine-glucuronide (Nor-BUP-g) after sublingual administration of 4 (red), 8 (green) and 16 mg (blue) of buprenorphine every 24 h (QD). (**B**) (Bottom) Buprenorphine steady state concentrations from 1000 virtual patients receiving 4 (red), 8 (green) and 16 mg (blue) of sublingual BUP every 24 h (QD)obtained with the population pharmacokinetic model. The dashed line represents the exposure threshold of 3 ng/mL. (**C**) Deterministic simulation of buprenorphine (BUP), nor-buprenorphine (Nor-BUP), buprenorphine-glucuronide (BUP-g) and nor-buprenorphine-glucuronide (Nor-BUP-g) after sublingual administration of 8 (light blue) and 16 mg (brown) of buprenorphine every 8 h (TID) or 12 h (BID), respectively. (**D**) Buprenorphine steady state concentrations from 1000 virtual patients receiving 4 (grey), 8 (light blue) and 16 (orange) mg of sublingual BUP every 6 (QID), 8 (TID) and 12 (BID) hours obtained with the population pharmacokinetic model.

Our study was the first to characterize not only the pharmacokinetics of BUP in a PR population diagnosed with OUD, but also to report BUP metabolite concentrations in this population. BUP is metabolized by *N*-dealkylation mediated primarily by CYP3A4 to Nor-BUP. Glucuronidation of BUP and Nor-BUP to BUP-g and Nor-BUP-g is mediated by UGT1A1 and UGT1A3 [32]. The CYP-mediated buprenorphine metabolic pathway to Nor-BUP and Nor-BUP-g of recruited subjects appears to predominate compared to UGT, as evidenced by the significantly higher Nor-BUP-g plasma levels relative to the other metabolites. Additionally, analyzing our *AUC*_0–8h_ estimates to measure CYP and UGT mediated BUP metabolism, our Puerto Rican subjects had higher CYP-mediated *N*-methylation of BUP compared to reported values by Zhang during pregnancy in a White population, although similar UGT activity was found between both groups [18]. This observation points to potential differences in *N*-demethylation activity between the two populations. Specific reasons for such differences are not clear at this time. However, a preliminary analysis using the developed popPK model and a deterministic simulation of the metabolites after sublingual administration of 4, 8 and 16 mg of BUP (Figure 5A) shows a relevant accumulation of Nor-BUP-g in plasma. A covariate analysis was performed to assess the impact of age, body weight and gender on the parameters of the population PK model. No statistically significant relationship was detected that was able to characterize any covariate–parameter relationship that would help to partially explain the inter-individual variability observed. However, the low number of patients recruited may have influenced the conclusions of the covariate analysis, and further evaluations are encouraged with larger sample size studies.

The popPK model developed herein represents the first joint population analysis of BUP and its metabolites in steady-state OUD patients in PR. Although preliminary, the quantitative framework reached has allowed characterizing the metabolic process of BUP, together with the time course of its metabolites, in a satisfactory way. The model establishes a favorable sequential formation of Nor-BUP and Nor-BUP-g metabolite (*k*_12_). Others have suggested that the distribution of BUP occurs in two peripheral compartments [33,34,35]. However, due to sample size limitations and short sampling time, we were unable to accommodate a three-compartment model, perhaps because of the insufficient number of samples obtained at steady state until 8 h post-administration. Additionally, the majority of the patients received 8 mg dose, with only one patient receiving a 16 mg dose and three receiving a 4 mg dose. It is necessary to incorporate more patients at different dose levels, which would allow possible non-linear pharmacokinetic mechanisms to be addressed. Other population models have been proposed in recent years to characterize the BUP time-course in healthy volunteers and patients after IV, oral or SC administration of BUP, which partially differ from the population PK model we proposed herein. Initially, we intended to adapt the previous population PK models to characterize the experimental observations of BUP and its metabolites, but they failed to describe the overall performance of each analyte [12,13,35,36]. Knowing that our experimental information is limited, we adapted structures (peripheral distribution of BUP and buccal metabolism of BUP) and processes (first-order absorption and disposition) from previous models in order to maintain a theoretical framework similar to the previously proposed models. Additional plasma levels within a dosing interval (24 h) is required to allow for confirmation and/or adaptation of the model. Patients also need to be more closely monitored to avoid any additional dose administration throughout the sampling period. This should increase the estimating capabilities of the model much needed for accurate implementation in a clinical setting. Despite all the limitations, the BUP predictions obtained from the developed popPK model have been externally validated in a satisfactory way from data published in different population groups (Table 5). The validity of the proposed structural mechanisms has been corroborated, since 88% (*AUC*) and 97% (*C_max_*) of the ratios were within ±50%. The prediction intervals, represented by the 90% CI, show how the model is able to capture the mean values of BUP observed in the literature, possibly also as a consequence of the high variability observed. This preliminary model is, however, a first step in estimating BUP dosing strategies in any population group.

Trough BUP levels obtained in this study suggest that most of the recruited subjects may potentially experience withdrawal symptoms before taking their daily maintenance BUP dose, based on published data [11]. Most of the recruited subjects, either just before dosing or at the last blood draw, had blood levels below 3 ng/mL, which has been previously associated with withdrawal and negative opioid reinforcement effects [11]. Although our study did not collect clinical withdrawal data, we anecdotally observed some patients showing anxiety, irritability and restlessness just before taking their daily dose (at trough), even though patients were previously stabilized. It is important that pharmacodynamic assessments are performed to improve the understanding of BUP dose-related opioid receptor saturation in this population. In this sense, we have performed a stochastic simulation using our preliminary developed model in steady state conditions after the administration of 4, 8 and 16 mg of BUP. Although exploratory, this initial evaluation may estimate and potentially quantify the proportion of patients that would show a trough level above 3 ng/mL in order to preliminarily propose the optimal dose level to be administered in these patients. According to these initial results, 0%, 0% and 35% of the simulated patients taking 4, 8 and 16 mg, respectively, would show a steady state concentration (*C_ss_*) above the threshold limit of 3 ng/mL. These preliminary simulations suggest a high probability of withdrawal symptoms of patients receiving 4 and 8 mg of BUP (Figure 5B). Moreover, based on the population preliminary popPK model developed herein, two exploratory yet optimized BUP dosing schedules have been proposed able to achieve a 80% proportion of patients with *C_ss_* ≥ 3 ng/mL: 8 mg of BUP three times a day (TID) or 16 mg of BUP twice-daily (BID) predict a probability of 81 and 93%, respectively, of patients with *C_ss_* levels equal of greater than 3 ng/mL (Figure 5C,D). Simulations of a once-daily schedule provide a wider range of concentrations for each analyte, whereas the twice-daily schedule guarantees a lesser fluctuation in concentration levels for each analyte, which may be relevant to avoid any safety concern. Because this is an exploratory analysis, it is important that these approximations are re-evaluated and validated with accurate population modeling strategies. It is possible that the pharmacodynamics of BUP may be different in PR, and a lower trough may be sufficient. Future studies evaluating the pharmacodynamics of BUP in PR will need to address this. Evidence-based BUP dose adjustments to either increase the dose or the dosing frequency have been previously recommended during pregnancy to sustain plasma concentrations above the threshold of 1 ng/mL, and thus, prevent withdrawal symptoms and improve adherence [37].

In summary, this study presents a pilot description of BUP pharmacokinetics in an OUD Puerto Rican population treated with extrapolated dosing protocols from the US developed in the White patient populations groups. These protocols are not evidence-based, and thus, are not culturally relevant. Although our study had limitations such as sample size and sampling time (limited to 8 h), it provides an insight into specific BUP pharmacological characteristics in the population that should be addressed to improve the clinical outcomes of OUD care in PR using BUP. A refined pharmacologic modeling approach that assesses specific pharmacokinetic characteristics in the population is needed to accurately dose BUP in PR. A population pharmacokinetic analysis may help in incorporating population characteristics and investigate sources of variability. This approach will allow the understanding of the intrinsic variability in exposure and pharmacotherapy outcomes in relation to OUD treatment effectiveness in a Puerto Rican population.

## 5. Conclusions

In summary, this study presents for the first time a description of the pharmacokinetics of BUP and its metabolites in an OUD population in PR. Despite the limitations, our study provides insight into specific BUP pharmacokinetic characteristics and the metabolic processes involved after its administration. The population PK model, which has been externally validated using published data across several studies, is a preliminary framework able to describe the time-course of BUP, BUP-g, Nor-BUP and Nor-BUP-g. This study also proposed a model-informed dose selection strategy that suggests dose frequency adjustments to decrease the risk of withdrawal symptoms in the PR population.

## Figures and Tables

**Table 1:** Buprenorphine non-compartmental pharmacokinetic estimates presented as mean, standard deviation (SD) and relative standard deviation (RSD).

BUP PK	Dose ^1^ (mg)	*t_max_* (h)	*C_max_* (ng/mL)	*AUC*^2^ (ng·h/mL)	Dose Normalized	
					*C_max_* (ng/mL)/mg	*AUC* (ng·h/mL)/mg
Mean	7.64	1.45	7.18	26.80	1.06	3.84
SD	3.32	0.69	5.76	17.87	0.79	2.35
RSD	43%	47%	80%	66%	74%	61%

^1^ Dose administered the day of the study, used for dose-adjustment calculations. ^2^ AUC corresponding to 0 to 8 h; sampling period. SD: standard deviation; RSD: relative standard deviation.

**Table 2:** Mean observed maximum and minimum concentration of buprenorphine (BUP), norbuprenorphine (Nor-BUP), buprenorphine-3-glucuronide (BUP-g) and norbuprenorphine-3-glucuronide (Nor-BUP-g).

BUP PK	*C_max_* (ng/mL)				*C_min_*^2^ (ng/mL)			
	BUP ^1^	Nor-BUP	BUP-g	Nor-BUP-g	BUP	Nor-BUP	BUP-g	Nor-BUP-g
Mean	7.18	3.56	7.58	16.32	1.67	1.60	0.72	9.11
SD	5.76	4.9	10.6	19.7	1.44	2.03	1.10	11.61

^1^ Same as presented in Table 1; ^2^ Trough value (*C_o_*) was taken as the minimum concentration; SD: standard deviation.

**Table 3:** Mean buprenorphine *AUC* ratios of CYP and UGT mediated metabolic pathways.

	CYP ^1^	UGT ^2^
	*AUC*_nBup+nBupG_/*AUC*_Bup_	*AUC*_BupG_/*AUC*_Bup_
Mean	3.34	0.39
SD	1.87	0.26
RSD	55%	66%

^1^ CYP mediated *N*-demethylation buprenorphine metabolic pathway; ^2^ UGT activity mediating buprenorphine metabolism; SD: standard deviation; RSD: relative standard deviation.

**Table 4:** Final population pharmacokinetic parameter estimates after sublingual buprenorphine administration in patients. RSE: relative standard error.

Parameter		Units	Final PK Model		Bootstrap Analysis (*n* = 500)		
			Value	RSE [%]	Median	2.5th	97.5th
Fixed-effects	K12	[1/h]	2.34	34	2.41	0.77	4.11
	K14	[1/h]	1.29	61	1.27	0.84	3.98
	K24	[1/h]	1.28 × 10^−1^	32	1.19 × 10^−1^	7.42 × 10^−2^	1.54 × 10^−1^
	K23	[1/h]	6.43 × 10^−2^	36	6.32 × 10^−2^	2.69 × 10^−2^	1.07 × 10^−1^
	K26	[1/h]	2.18 × 10^−1^	23	2.11 × 10^−1^	1.12 × 10^−1^	3.41 × 10^−1^
	K62	[1/h]	4.10 × 10^−2^	14	4.21 × 10^−2^	3.86 × 10^−2^	4.61 × 10^−2^
	K35	[1/h]	1.23 × 10^−1^	52	1.31 × 10^−1^	7.41 × 10^−2^	2.65 × 10^−1^
	K45	[1/h]	8.73 × 10^−1^	43	8.81 × 10^−1^	4.55 × 10^−1^	1.31
	K30	[1/h]	3.81 × 10^−4^	21	3.62 × 10^−1^	3.11 × 10^−3^	4.97 × 10^−3^
	K50	[1/h]	1.27 × 10^−1^	15	1.39 × 10^−1^	9.91 × 10^−2^	1.54 × 10^−1^
	KA	[1/h]	2.54	58	2.43	1.71	4.38
	V2/F	[L]	861	11	872	811	965
	V3/F	[L]	702	16	712	623	802
Inter-individual variability	K24	[%]	41	87	43	15	87
	K23	[%]	63	78	65	11	76
	K35	[%]	70	69	71	38	134
	K45	[%]	56	121	54	13	159
	K50	[%]	54	38	58	21	64
Residual error	BUP	[%]	38	18	35	22	48
	Nor-BUP	[%]	29	21	26	13	39
	BUP-g	[%]	80	61	78	42	187
	Nor-BUP-g	[%]	26	41	25	12	38

**Table 5:** External validation of model-derived exposure endpoints (*AUC* and *C_max_*) using the population pharmacokinetic model in the dose range from 2 to 32 mg after sublingual administration of buprenorphine in a single dose regimen.

Doses (mg)	Predicted *AUC* (ng·h/mL)			Observed *AUC* (ng·h/mL)	*AUC* Ratio	Predicted *C_max_* (ng/mL)			Observed *C_max_* (ng/mL)	*C_max_* Ratio	Reference
	5th	95th	Mean	Mean		5th	95th	Mean	Mean		
16	31.56	71.84	54.13	45.64	1.19	6.15	8.27	7.47	6.25	1.20	M. Jönsson, European Journal of Pharmaceutical Sciences 122 (2018) 125–133; [21]
4	8.54	22.87	15.86	17.84	0.89	1.91	3.54	2.71	2.44	1.11	
4	8.54	22.87	15.86	12.52	1.27	1.91	3.54	2.71	1.84	1.47	Harris et al. Healthy females and male non-opioid-dependent users, age range (22–42 years); [22]
8	19.11	40.34	29.74	20.22	1.47	2.51	5.27	3.87	3	1.29	
16	31.56	71.84	54.13	34.89	1.55	6.15	8.27	7.47	5.95	1.26	
16	31.56	71.84	54.13	32.63	1.66	6.15	8.27	7.47	5.47	1.37	
2	-	-	-	-	-	0.89	1.72	1.12	1.06	1.06	McAleer et al. Opioid-naïve healthy male subjects, age range (19–42); [23]
8	19.11	40.34	29.74	31.81	0.93	2.51	5.27	3.87	4	0.97	
12	29.63	54.22	42.59	41.61	1.02	4.29	7.61	5.84	5.4	1.08	
16	31.56	71.84	54.13	52	1.04	6.15	8.27	7.47	6.4	1.17	
8	19.11	40.34	29.74	24.55	1.21	2.51	5.27	3.87	3.2	1.21	
8	19.11	40.34	29.74	24.6	1.21	2.51	5.27	3.87	3.2	1.21	
4	8.54	22.87	15.86	9.37	1.69	1.91	3.54	2.71	2	1.36	Ciraulo et al. Healthy non-opioid-dependent users, age range (21–45); [24]
8	19.11	40.34	29.74	19.92	1.49	2.51	5.27	3.87	2.65	1.46	
16	31.56	71.84	54.13	34.94	1.55	6.15	8.27	7.47	4.42	1.69	
24	51.93	82.74	67.56	48.81	1.38	7.95	10.67	9.44	5.41	1.74	
4	8.54	22.87	15.86	13.09	1.21	1.91	3.54	2.71	2.33	1.16	Ciraulo et al. Healthy non-opioid-dependent users, age range (21–55); [23]
8	19.11	40.34	29.74	23.23	1.28	2.51	5.27	3.87	3.53	1.10	
16	31.56	71.84	54.13	39.38	1.37	6.15	8.27	7.47	5.83	1.28	
24	51.93	82.74	67.56	47.55	1.42	7.95	10.67	9.44	6.44	1.47	
16	31.56	71.84	54.13	54.7	0.99	6.15	8.27	7.47	6.88	1.09	Compton et al. Healthy female and male ipioi-dependent-users, age range (18–65); [25]
24	51.93	82.74	67.56	81.1	0.83	7.95	10.67	9.44	9.1	1.04	
32	68.49	102.37	84.31	103	0.82	12.41	16.29	14.21	13.93	1.02	
2	5.14	9.63	7.41	6.5	1.14	0.89	1.72	1.12	0.85	1.32	Greenwald et al. Healthy female and male opioid-dependent-users, age range (34–45); [10]
16	31.56	71.84	54.13	41.9	1.29	6.15	8.27	7.47	5.15	1.45	Moody et al. Drug Alcohol Depend. (2011) 118(2–3), 479–483; [15]
16	31.56	71.84	54.13	58.4	0.93	6.15	8.27	7.47	6.97	1.07	
2	5.14	9.63	7.41	10.9	0.68	0.89	1.72	1.12	1.65	0.68	R. Dong et al. Drugs in R&D (2019) 19, 255–265; [14]
4	8.54	22.87	15.86	18.1	0.88	1.91	3.54	2.71	2.57	1.05	
8	19.11	40.34	29.74	33.3	0.89	2.51	5.27	3.87	5	0.77	
12	29.63	54.22	42.59	47.7	0.89	4.29	7.61	5.84	7.03	0.83	
16	31.56	71.84	54.13	55.6	0.97	6.15	8.27	7.47	7.84	0.95	
24	51.93	82.74	67.56	73	0.93	7.95	10.67	9.44	11.7	0.81	
4	8.54	22.87	15.86	23.89	0.66	1.91	3.54	2.71	3.31	0.82	Kuhlman et al. Journal of Analitical Toxicology Vol 20 Oct 1996, p369; [26]
2	5.14	9.63	7.41	6.789	1.09	0.89	1.72	1.12	0.78	1.44	Healthy adult male or non-pregnant, non-breastfeeding female volunteers, 18–45 years of age (inclusive), BMI between 18 and 30 kg/m^2^ (inclusive) and weighed a minimum of 50 kg (110 lbs); [27]

## Supplementary Materials

The following are available online at https://www.mdpi.com/1999-4923/12/12/1226/s1, Figure S1: Standard goodness of fit plots. Red, orange, green, and blue dots represent buprenorphine, nor-buprenorphine, buprenorphine-glucuronide, and nor-buprenorphine-glucuronide. IWRES: individual weighted residuals. CWRESI: conditional weighted residuals. Grey line represents the regression line using the loess method. Dotted blue line indicates the identity line (upper plots) or the reference limits of a Gaussian distribution. Figure S2: Numerical Prediction Distribution Error plots (NPDE). LN PRED: log-transformed population predictions. Grey line represents the regression line using the loess method. Dotted blue line indicates the identity line (upper plots) or the reference limits of a Gaussian distribution. Figure S3: Individual prediction plot. Red, orange, green, and blue dots represent the experimental buprenorphine, nor-buprenorphine, buprenorphine-glucuronide, and nor-buprenorphine-glucuronide observations. Grey line represents individual predicted concentrations obtained with the population PK model.
