# Supplementary material for: Pharmacokinetic Characterization and External Evaluation of a Quantitative Framework of Sublingual Buprenorphine in Patients with an Opioid Disorder in Puerto Rico
Source: Pharmaceutics. 2020 Dec 18;12(12):1226. doi: 10.3390/pharmaceutics12121226 (PMC7766849; doi:10.3390/pharmaceutics12121226)
Supplement: Supplementary file 1 [file pharmaceutics-12-01226-s001.pdf]

pharmaceutics

Supplementary Materials: Pharmacokinetic Characterization and External Evaluation of a Quantitative Framework of Sublingual Buprenorphine in Patients with an Opioid Disorder in Puerto Rico

Darlene Santiago, Victor Mangas-Sanjuan, Kyle Melin, Jorge Duconge, Wenchen Zhao and Raman Venkataramanan

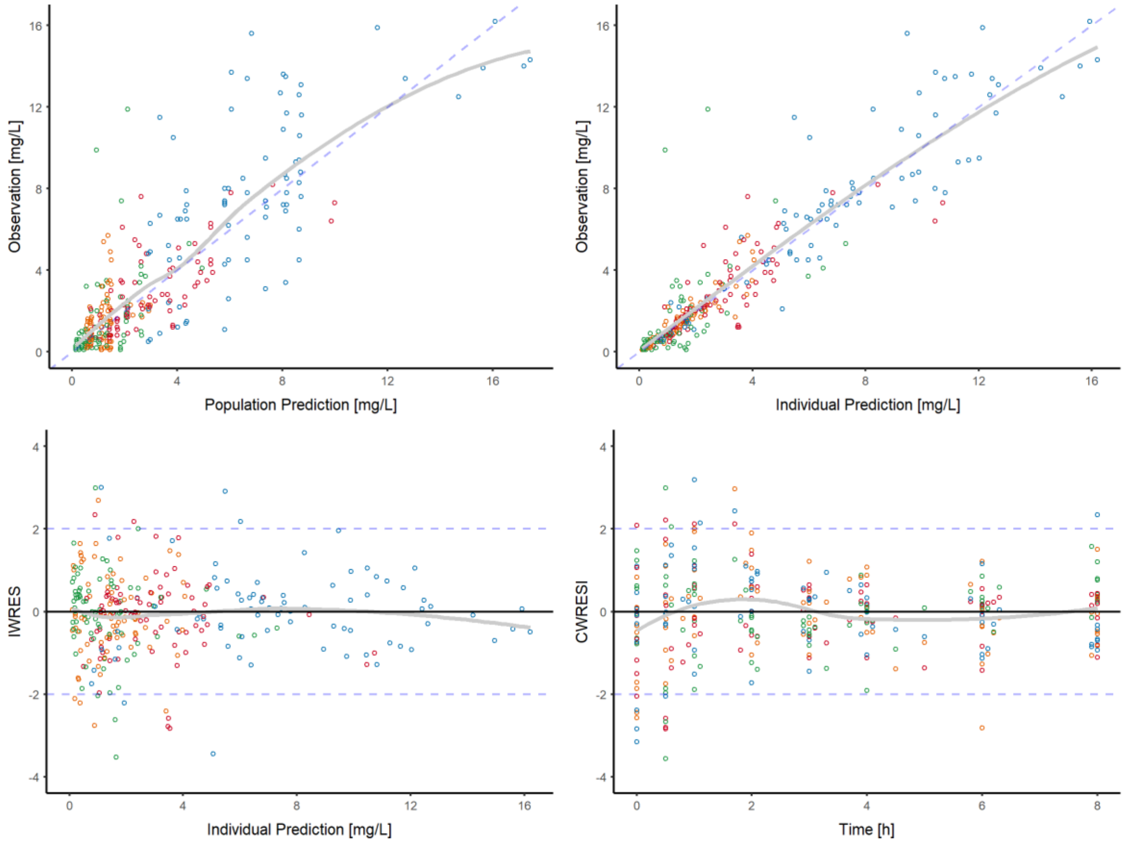

Figure S1. Standard goodness of fit plots. Red, orange, green, and blue dots represent buprenorphine, nor-buprenorphine, buprenorphine-glucuronide, and nor-buprenorphine-glucuronide. IWRES: individual weighted residuals. CWRESI: conditional weighted residuals. Grey line represents the regression line using the loess method. Dotted blue line indicates the identity line (upper plots) or the reference limits of a Gaussian distribution.

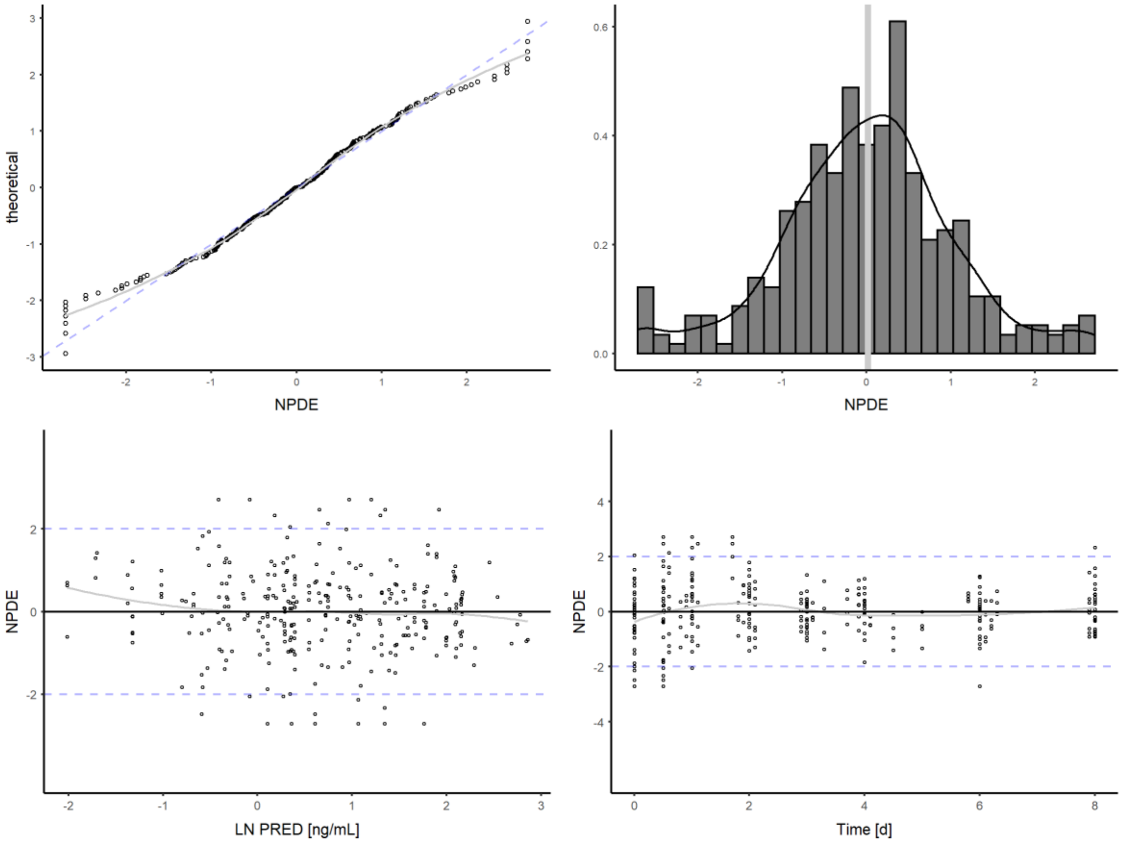

Figure S2. Numerical Prediction Distribution Error plots (NPDE). LN PRED: log-transformed population predictions. Grey line represents the regression line using the loess method. Dotted blue line indicates the identity line (upper plots) or the reference limits of a Gaussian distribution.

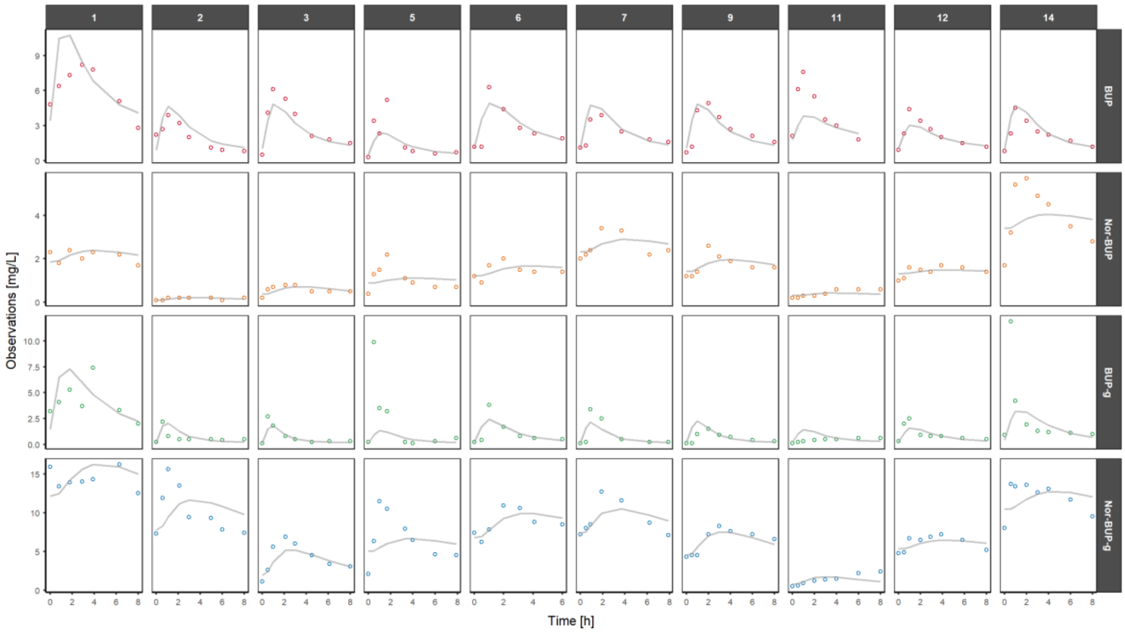

Figure S3. Individual prediction plot. Red, orange, green, and blue dots represent the experimental buprenorphine, nor-buprenorphine, buprenorphine-glucuronide, and nor-buprenorphine-glucuronide observations. Grey line represents individual predicted concentrations obtained with the population PK model.
